# Long read sequencing revealed proventricular virome of broiler chicken with transmission viral proventriculitis

**DOI:** 10.1186/s12917-022-03339-9

**Published:** 2022-06-29

**Authors:** Tianxing Yan, Gen Li, Defang Zhou, Liping Hu, Xiaojing Hao, Ruiqi Li, Guihua Wang, Ziqiang Cheng

**Affiliations:** 1 Present Address: College of Veterinary Medicine, Shandong Agricultural University, Shandong Provence, Tai’an, 271018 China; 2 College of Veterinary Medicine, Qingdao Agricultural University, Qingdao, 266000 China; 3 Animal Epidemic Prevention and Control Center of Shandong Province, Jinan, China; 4 Animal Husbandry and Veterinary Research Institute of Qingdao, Qingdao, China

**Keywords:** Transmissible viral proventriculitis, Virome, Gyrovirus, Novel CRESS virus, Pacific Biosciences RSII platform

## Abstract

**Background:**

Transmissible viral proventriculitis (TVP) causes significant economic loss to the poultry industry. However, the exact causative agents are obscure. Here we examine the virome of proventriculus from specified pathogen free (SPF) chickens that reproduced by infection of proventricular homogenate from broiler chicken with TVP using long read sequencing of the Pacific Biosciences RSII platform. The normal SPF chickens were used as control.

**Results:**

Our investigation reveals a virome of proventriculitis, including three Gyrovirus genera of the *Aneloviridae*: Gyrovirus homsa1 (GyH1) (also known as Gyrovirus 3, GyV3) (n = 2662), chicken anemia virus (CAV) (*n* = 482) and Gyrovirus galga1 (GyG1) (also known as avian Gyrovirus 2, AGV2) (*n* = 11); a plethora of novel CRESS viral genomes (*n* = 26) and a novel genomovirus. The 27 novel viruses were divided into three clusters. Phylogenetic analysis showed that the GyH1 strain was more closely related to the strains from chicken (MG366592) than mammalian (human and cat), the GyG1 strain was closely related to the strains from cat in China (MK089245) and from chicken in Brazil (HM590588), and the CAV strain was more closely related to the strains from Germany (AJ297684) and United Kingdom (U66304) than that previously found in China.

**Conclusion:**

In this study, we revealed that Gyrovirus virome showed high abundance in chickens with TVP, suggesting their potential role in TVP, especially GyH1. This study is expected to contribute to the knowledge of the etiology of TVP.

**Supplementary Information:**

The online version contains supplementary material available at 10.1186/s12917-022-03339-9.

## Background

Transmissible viral proventriculitis (TVP) is an infectious disease reported in all types of chickens and has significant impact on the poultry industry. The typical pathological lesions of TVP are necrosis of oxynticopeptic cells, lymphocytic infiltration and hyperplastic ductal epithelium which replaces the glandular epithelium [1]. Due to the lesions in the proventriculus, affected birds suffer from maldigestion, poor feed conversion and stunted growth [2].

The first case of TVP was reported by Kouwenhoven in 1978 in Netherlands [3]. They reported a case of proventriculitis in commercial chicken broilers and proved that TVP was induced by an infectious factor. Since then, TVP cases have been identified and reported worldwide. However, the etiology of TVP has not been explicitly defined so far. Several studies have supported the association between chicken proventricular necrosis virus (CPNV) and TVP [4]. CPNV was detected in commercial broiler chickens as an adenovirus-like virus (R11/3 strain) for the first time in the USA [5]. Consequently, the R11/3 strain virus was identified as Birnavirus, and named CPNV [6, 7]. Since then, CPNV was reported in the USA, Spain, France, UK, Poland and Brazil [8–13]. Studies on TVP etiopathology also imply the other viruses involvement, including infectious bursal disease viruses (IBDV) of the *Birnaviridae* family, infectious bronchitis viruses (IB) of the *Coronaviridae* family, reoviruses (REO), picornaviruses, fowl adenoviruses (FAdV), adeno-like viruses, Gyrovirus homsa1 (GyH1, also known as Gyrovirus 3, GyV3), Cyclovirus (CyCV) of the *Anelloviridae* [14, 15] and chicken Circovirus (CCV) of the *Circoviridae* [16].

To identify the definite viruses of TVP, many researchers employed metagenomic analysis, such as next generation sequencing (NGS) technology [17]. However, NGS has several serious limitations such as short read lengths (approximately 400 bp) and amplification biases [18]. Moreover, some viruses have high CG content regions, which is generally challenging to amplify and thus poorly resolved by short-read sequencing. These factors restrict our ability to understand the real landscape of the real viral genomes in TVP.

Recently, a novel long reads third-generation sequencing (TGS) technology, single molecular real-time (SMRT) sequencing using the PacBio RSII, has been developed [19, 20]. The advantages of TGS is the long-read length that allows for greater certainty in read overlap and assembly, thus providing better resolution of repetitive regions and structural variants [21]. The average sequencing read length from the current PacBio RSII instrument is about 12 kbp. The technology has been applied to various research such as determination of the full-length genome sequences of bacteria, viruses, haplotype genes, variant transcriptome or human genes [22, 23].

In this study, we collected TVP cases from broiler flocks, which showed significant stunted growth and were examined by histopathology, and were PCR negative for infectious bursa disease virus (IBDV), avian leukosis virus subgroup J (ALV-J), reticuloendotheliosis virus (REV), Marek’s disease virus (MDV), infectious bronchitis virus (IBV), reovirus (ReoV) and fowl adenovirus 4 (FAdV-4). The supernatant of proventricular homogenate was inoculated into one-day-old specified pathogen free (SPF) chicks to observe the process of TVP. At 21dpi, the infected chickens showed typical TVP, and the supernatant of proventriculi homogenate was pooled, extracted DNA/RNA, and performed long-read sequencing by PacBio RSII platform, and the same day old SPF chickens were used as control.

## Materials and methods

### Sample preparation

Chickens are diagnosed with TVP based on the clinic symptoms, gross and histological lesions of the proventriculus, such as, stunted growth, slightly enlarged proventriculus, necrosis of oxynticopeptic cells, lymphocytic infiltration and hyperplastic ductal epithelium which replaces the glandular epithelium. Thirty broiler chickens with TVP between 10 and 35 days of age were collected from five Chinese farms. Chickens were only present with lymphocytic proventriculitis, and PCR was negative for CPNV, IBDV, ALV-J, REV, MDV, IBV, ReoV and FAdV-4.

To reproduce TVP in SPF chickens, the supernatant of ten proventriculi homogenates from broiler chickens with TVP were pooled and used to reproduce TVP in SPF chickens. Briefly, twenty one-day-old SPF chicks were intraperitoneally inoculated with 1 mL supernatant of proventriculi homogenates from broiler chickens with TVP, and ten one-day-old SPF chicks were intraperitoneally inoculated with PBS as control. They reared in SPF chicken isolators to prevent exogenous bacteria/viruses. Three chickens of the infected group and two chickens of the control group were euthanized and necropsied at 7, 14, 21, 28 and 35 dpi. After sacrifice, a complete necropsy was performed and histology lesions were observed. Fifteen proventriculi were collected from chickens with TVP or ten control chickens for sequencing sample preparation. Briefly, 3 g proventricular tissues was sheared from SPF chickens with TVP and homogenized in 12 mL phosphate buffered saline (PBS), and then centrifugated at 2400 × *g*, 4 °C for 30 min. The supernatant was transferred to a fresh tube and centrifugated at 5,000 × *g*, 4 °C for 15 min. The supernatant was removed and filtered through sterile 0.22 µm syringe filters (Sartorius). The filtered supernatant was then ultracentrifuged (Eppendorf) for 5 h at 113,000 × *g*, 4 °C. The supernatant was removed and the pellet was resuspended in 1 mL sterile PBS buffer, pH 7.2.

### Total DNA and RNA extraction

To remove the exogenous nucleic acids, RNase A and DNase 1 (100 U) (Thermo Scientific) were added according to the manufacturer's guidelines. The suspension was incubated in a water bath at 37 °C for 30 min followed by inactivation by adding ethylenediaminetetraacetic acid (EDTA) (Thermo Scientifific), and incubated at 65 °C for 10 min. Samples were divided into two fractions for DNA and RNA extraction and placed on ice.

Total RNA was extracted from samples as prepared above using the Ribopure RNA extraction kit (Qiagen) according to the manufacturer’s guidelines, and then RNA integrity was detected by agarose gel electrophoresis for 28 s and 18 s brightness ratio. The purity of RNA was confirmed using Nano Drop (Thermo Fisher Scientific, MA, USA) that revealed an 260/280 ratio from 1.9 to 2.2. RNA was reverse transcribed into cDNA by the RNA Reverse Transcription kit (Qiagen). The process was 42℃ 15 min, reverse transcription reaction; 95℃ 3 min, enzyme inactivation. Total DNA was extracted using the Viral DNA Mini Kit (Qiagen) according to the manufacturer’s guidelines. To produce enough genomic material for sequencing, the cDNA and DNA were mixed at 1:1 together for whole genome amplification (WGA). WGA reactions was carried out using the Repli-g Cell WGA and WTA Kit (Qiagen) according to the manufacturer’s guidelines using random primers.

### Sequencing and data analysis

The library for single-molecule real-time (SMRT) sequencing was constructed with an insert size of 20 kb using the SMRT bell TM Template kit (version 1.0). Briefly, the process were that fragment and concentrate DNA, repair DNA damage and ends, prepare blunt ligation reaction, purify SMRTbell Templates with 0.45X AMPure PB Beads, size-selection using the BluePippin System, repair DNA damage after size-selection. At last, the library quality was assessed on the Qubit® 2.0 Fluorometer (Thermo Scientific) and detected the insert fragment size by Agilent 2100(Agilent Technologies). Sequencing reactions were performed by the PacBio Sequel sequencer (BGI-Shenzhen, China) with Sequel Sequencing Kit 2.1.

All the raw data were trimmed by SOAPnuke v.1.5.2. The trimmed reads were mapped to the host genome using SOAP2 software to identify and remove host originated reads. The quality of generated sequences was evaluated using FastQC tool [24]. Both reads and generated contigs were subjected to blastx with GenBank database (https://www.ncbi.nlm.nih.gov, last accessed on 10 August 2020). Only those with an e-value lower than 10^−5^ were considered [25]. The contigs were analyzed and annotated using Geneious v. 8.1.3 software. The contings were assembled by the SMRT Link software (v5.0.1), the arrow algorithm was used to correct and count the variant sites in the preliminary assembly results.

Genome organization and sequence analysis was performed using the SnapGene viewer software. Multiple sequence alignment was performed using the Clustal W program, and the comparison of sequence identity was performed using MegAlign software (DNAStar). Phylogenetic analysis of genes was performed using MEGA 7.0 using the neighbor-joining method with 1,000 bootstrap replicates.

## Results and discussion

To confirm the TVP in broiler chickens and avoid the interference of unnecessary factors, we reproduced TVP in SPF chicks. The clinical manifestations, gross and histology lesions of infected chicks were the same as previous report (Supplementary Figure) [26]. Fifteen proventriculi from infected chickens and ten proventriculi from control chickens were pooled and sampled, respectively.

**Fig. 1:**
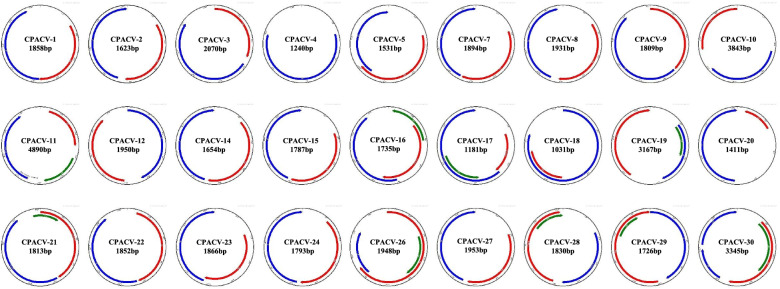
Genome organization of 26 novel CRESS virus and one Genomovirus. CAPCV is the temporary name for these viruses. The direction, relative size, and reading frame of each Open Reading Frame (ORF) are indicated by the location, length, and color, respectively, of the arrows located internally to the virus genomic. Red, blue and green represent replication-associated protein (Rep), represents capsid protein (Cap) and VP3, respectively. All ORF are predicted using the SnapGene viewer software

Using the PacBio RSII platform, we identified 3193 haplotype complete genomes of single-stranded DNA (ssDNA) viruses from 15 proventriculi samples of SPF chickens with TVP. Of these, 2662 genomes (83.37%) were Gyrovirus homsa1(GyH1) (also known as Gyrovirus 3, GyV3), 482 genomes (15.10%) were chicken anemia virus (CAV), and 11 genomes (0.34%) were Gyrovirus galga1 (GyG1) (also known as avian Gyrovirus 2, AGV2). 26 genomes were unique unclassified novel CRESS DNA viruses and a novel Genomovirus (Fig. 1). The three viruses of GyH1, CAV and GyG1 belong to Gyrovirus genus of *Anelloviridae.* They were present together in the form of haplotype virome in proventriculitis for the first time, indicating the high correlation between the virome and proventriculitis. The detail information of the 27 novel viruses was shown in Table 1. Among them, 59.26% (16/27) viruses were associated with environment, 3.7% (1/27) viruses (genomovirus, MW237851) were associated with plants, and the rest of them (10/27) were associated with animal tissues or feces. Three of the novel CRESS DNA viruses (MT975440, MW237843, MW237845) have a similar genome organization, i.e., their CP is encoded on the virion sense and their Rep on the complementary sense. The rest of novel CRESS DNA viruses genome organization showed clockwise sense.

**Table 1:** Identified 26 novel CRESS virus and a Genomovirus in proventriculus of broiler chickens with TVP

Number	Viral Classification	Complete genome	Temporary name	GenBank accession no	Homology virus	Accession no of homology virus	Identity	Source	GC%
1	CRESS virus	1858	CPACV 1	MT951397	putative replicase	AXH75828.1	39%	animal metagenome	45.86
2	CRESS virus	1623	CPACV 2	MT975438	Lake Sarah-associated circular virus-22	YP_009237603.1	45%	Lake water	46.77
3	CRESS virus	2070	CPACV 3	MT975439	Lake Sarah-associated circular virus-12	YP_009237573.1	38%	Lake water	38.84
4	CRESS virus	1240	CPACV 4	MT975440	capsid protein	AWW06122.1	38%	mouse tissue	38.39
5	CRESS virus	1531	CPACV 5	MT975441	Lake Sarah-associated circular virus-22	YP_009237602.1	46%	Lake water	42.65
6	CRESS virus	1894	CPACV 7	MW001861	Lake Sarah-associated circular virus-25	YP_009237609.1	38%	Lake water	44.03
7	CRESS virus	1931	CAPCV 8	MW001862	putative Rep	AUM61779.1	31%	wastewater	42.31
8	CRESS virus	1809	CAPCV 9	MW001863	putative replicase	AXH74921.1	34%	animal metagenome	42.29
9	CRESS virus	3843	CPACV 10	MW001864	capsid protein	AQU11778.1	46%	Sphagnum-dominated peatlands	30.39
10	CRESS virus	4890	CPACV 11	MW001865	capsid protein	AXH76335.1	40%	macaque stool	35.26
11	CRESS virus	1950	CAPCV 12	MW001866	capsid protein	AUM61702.1	26%	animal metagenome	32.72
12	CRESS virus	1654	CAPCV 14	MW237841	capsid protein	AYP28960.1	58%	animal metagenome	47.1
13	CRESS virus	1787	CAPCV 15	MW237842	capsid protein	AYP28960.1	58%	animal metagenome	43.93
14	CRESS virus	1735	CAPCV 16	MW237843	Rep	AYP28961.1	70%	crucian tissue	51.76
15	CRESS virus	1181	CAPCV 17	MW237850	capsid protein	AUM61760.1	32%	wastewater	41.32
16	CRESS virus	1031	CAPCV 18	MW237844	Rep	AYP28914.1	30%	animal metagenome	40.54
17	Genomovirus	3167	CAPCV 19	MW237851	Rep	QCS35885.1	32%	Capybara	41.36
18	CRESS virus	1411	CPACV 20	MW237845	capsid protein	AXH74652.1	35%	animal metagenome	44.65
19	CRESS virus	1813	CPACV 21	MW237852	Lake Sarah-associated circular virus-25	YP_009237609.1	38%	Lake water	43.19
20	CRESS virus	1852	CPACV 22	MW237846	Lake Sarah-associated circular virus-12	ALE29613.1	29%	Lake water	40.17
21	CRESS virus	1866	CPACV 23	MW237847	Lake Sarah-associated circular virus-24	YP_009237609.1	38%	Lake water	42.82
22	CRESS virus	1793	CPACV 24	MW237848	Rep	AUM61918.1	62%	environmental samples	43.56
23	CRESS virus	1948	CAPCV 26	MW237853	Lake Sarah-associated circular virus-23	YP_009237604.1	62%	Lake water	48.41
24	CRESS virus	1953	CPACV 27	MW237849	capsid protein	AUM61778.1	58%	environmental samples	40.91
25	CRESS virus	1830	CPACV 28	MW237854	capsid protein	AUM61778.1	58%	environmental samples	44.81
26	CRESS virus	1726	CAPCV 29	MW237855	Rep	AUM61966.1	45%	wastewater	47.8
27	CRESS virus	3345	CPACV 30	MW237856	Lake Sarah-associated circular virus-14	AIF34806.1	34%	Lake water	50.16

Identity: The homology with sequence in NCBI database

**Fig. 2:**
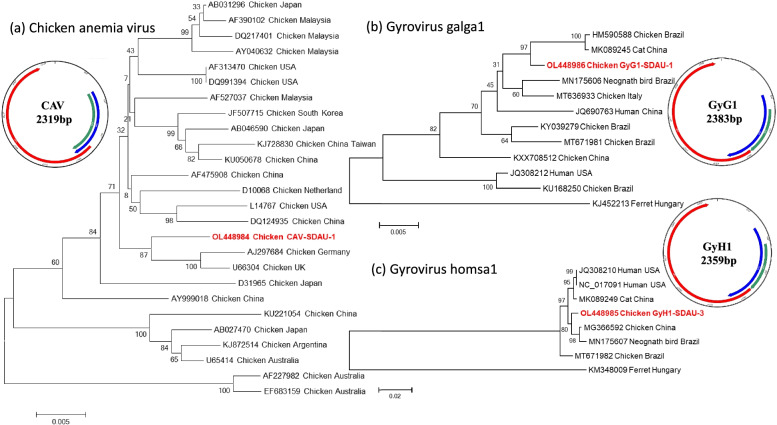
Genome organization and phylogenetic analysis of three gyroviruses. **a** Chicken anemia virus (CAV); **b** Gyrovirus galga1 (GyG1); **c** Gyrovirus homsa1 (GyH1). The reference sequences of other gyrovirus strains were obtained from GenBank database. Bootstrap values are presented at key nodes, used to evaluate the credibility of the branch. The bar indicates genetic distance. The virus strains identified in the study are shown with red

Phylogenetic analysis showed that the CAV strain (OL448984) was more closely related to the strains from Germany (AJ297684) and the United Kingdom (U66304) than viruses previously found in China (Fig. 2a) [27, 28]. This newly emerging CAV strains in China may originated from European countries, and the global circulation of commercial broilers has accelerated the spread of CAV. The GyG1 (OL448986) was closely related to the strains from cats in China (MK089245) and from chicken in Brazil (HM590588) (Fig. 2b) [29]. GyG1 found in cat feces may be derived from chickens consumed during the diet, chicken may be the original host of GyG1. The GyH1 (OL448985) was more closely related to the strains from chicken (MG366592) than mammalian (human and cat) (Fig. 2c) [14]. This suggested that GyH1 had a certain host specificity and it seemed very likely that GyH1 may, in fact, be a virus of avian origin. Overall, the three species GyVs were identified in proventriculus of chickens with TVP, indicating gyroviruses are highly resistant to gastric acid, which gives them the ability to persist and spread in the environment [30].

The diversity of proventriculitis lesions indicated that multiple viruses play a role in TVP. Several other viruses, including adenovirus [1], picornavirus [17] and CPNV [10], have been reported to be associated with TVP, although none of them could be definitively proved as cause of TVP. In this study, we revealed a new virome associated with TVP. This provides further insight into the true causes of TVP and future effective prevention and control of the disease.

## Conclusions

In this study, we reproduced the TVP in SPF chickens, and using long read sequencing in Pacbio-RSII sequencing platform to investigate the real etiology of TVP. The results revealed that Gyrovirus virome showed high abundance in chickens with TVP, suggesting their potential role in TVP, especially GyH1. This study is expected to contribute to the knowledge of the etiology of TVP.

## Supplementary Information

**Additional file 1.** Supplementary figure.

## Data Availability

The datasets generated and/or analysed during the current study are available in the National Center for Biotechnology Information (NCBI) repository, under these GenBank accession numbers MT951397, MT975438, MT975439, MT975440, MT975441, MW001861, MW001862, MW001863, MW001864, MW001865, MW001866, MW237841, MW237842, MW237843, MW237850, MW237844, MW237851, MW237845, MW237852, MW237846, MW237847, MW237848, MW237853, MW237849, MW237854, MW237855, MW237856, OL448984, OL448985 and OL448986.
