# Supplementary figures and images for: Long read sequencing revealed proventricular virome of broiler chicken with transmission viral proventriculitis
Source: BMC Vet Res. 2022 Jun 29;18:253. doi: 10.1186/s12917-022-03339-9 (PMC9241223; doi:10.1186/s12917-022-03339-9)

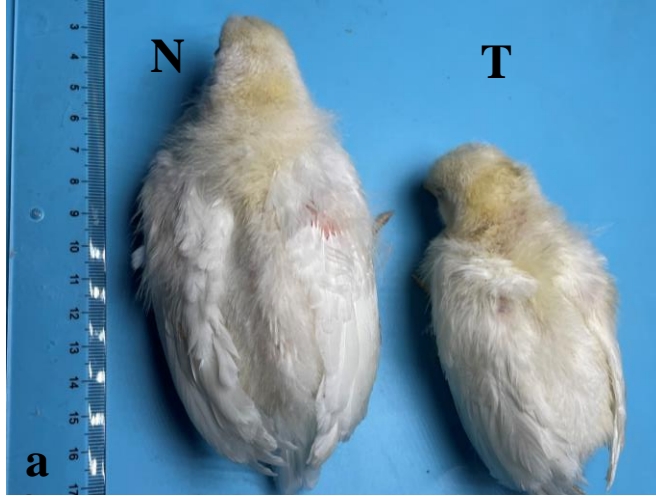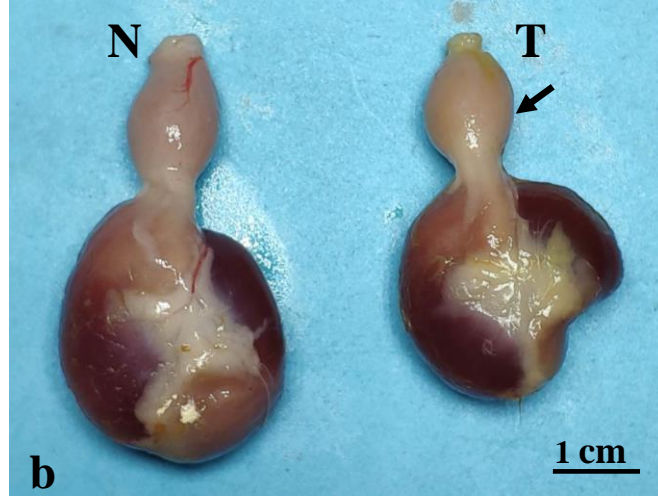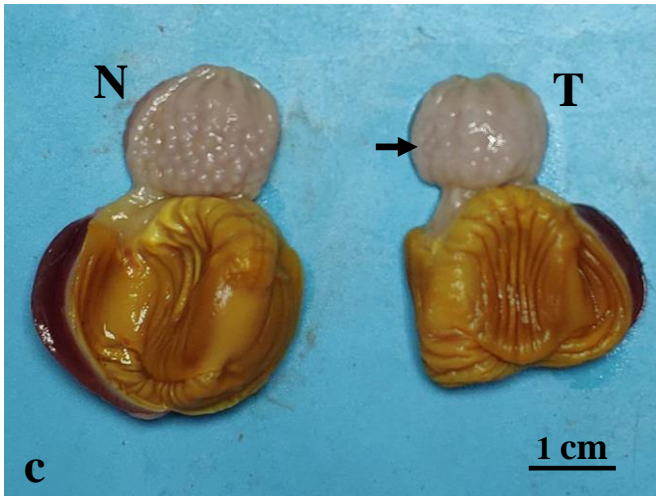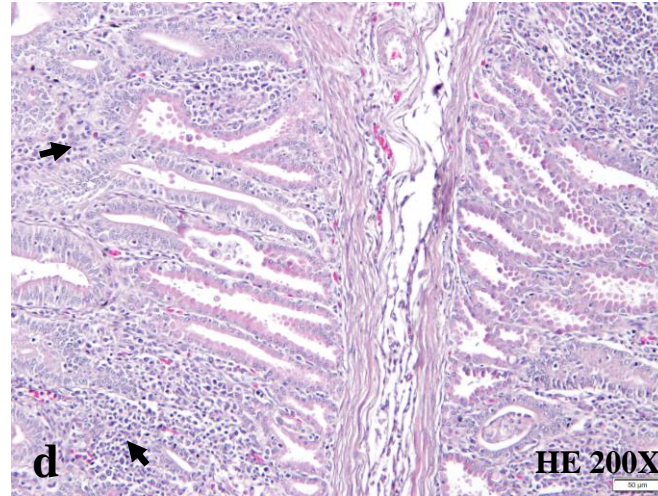

Supplement: Supplementary file 1 — Additional file 1. Supplementary figure. [file 12917_2022_3339_MOESM1_ESM.pdf]
